# Nivolumab versus chemotherapy in Japanese patients with advanced esophageal squamous cell carcinoma: a subgroup analysis of a multicenter, randomized, open-label, phase 3 trial (ATTRACTION-3)

**DOI:** 10.1007/s10388-020-00794-x

**Published:** 2020-11-10

**Authors:** Masanobu Takahashi, Ken Kato, Morihito Okada, Keisho Chin, Shigenori Kadowaki, Yasuo Hamamoto, Yuichiro Doki, Yutaro Kubota, Hisato Kawakami, Takashi Ogata, Hiroki Hara, Manabu Muto, Yuichiro Nakashima, Ryu Ishihara, Masahiro Tsuda, Satoru Motoyama, Mamoru Kodani, Yuko Kitagawa

**Affiliations:** 1 Department of Medical Oncology, Tohoku University Hospital, 1-1 Seiryo-machi, Aoba-ku, Sendai-shi, Miyagi, 980-8574 Japan; 2 Department of Gastrointestinal Medical Oncology, National Cancer Center Hospital, Tokyo, Japan; 3 Department of Surgical Oncology, Research Institute for Radiation Biology and Medicine, Hiroshima University, Hiroshima, Japan; 4 Department of Gastroenterological Chemotherapy, The Cancer Institute Hospital of Japanese Foundation for Cancer Research, Tokyo, Japan; 5 Department of Clinical Oncology, Aichi Cancer Center Hospital, Nagoya, Japan; 6 Keio Cancer Center, Keio University School of Medicine, Tokyo, Japan; 7 Department of Surgery, Osaka University Hospital, Osaka, Japan; 8 Department of Medicine, Division of Medical Oncology, Showa University School of Medicine, Tokyo, Japan; 9 Department of Medical Oncology, Kindai University Faculty of Medicine, Osakasayama, Osaka Japan; 10 Department of Gastrointestinal Surgery, Kanagawa Cancer Center, Yokohama, Japan; 11 Department of Gastroenterology, Saitama Cancer Center, Saitama, Japan; 12 Department of Clinical Oncology, Kyoto University Hospital, Kyoto, Japan; 13 Department of Surgery and Science, Graduate School of Medical Sciences, Kyushu University, Fukuoka, Japan; 14 Department of Gastrointestinal Oncology, Osaka International Cancer Institute, Osaka, Japan; 15 Department of Gastroenterological Oncology, Hyogo Cancer Center, Akashi, Japan; 16 Department of Thoracic Surgery, Akita University Graduate School of Medicine, Akita, Japan; 17 Department of Oncology, Ono Pharmaceutical Co., Ltd., Osaka, Japan; 18 Department of Surgery, Keio University School of Medicine, Tokyo, Japan

**Keywords:** Esophageal squamous cell carcinoma, Japanese population, Nivolumab, ATTRACTION-3

## Abstract

**Background:**

The efficacy and safety of nivolumab versus chemotherapy was evaluated in the Japanese subpopulation from the overall intent-to-treat (ITT) population of the ATTRACTION-3 trial conducted in patients with advanced esophageal squamous cell carcinoma (ESCC) as second-line treatment.

**Methods:**

Data from Japanese patients enrolled in the multicenter, randomized, open-label, phase 3 ATTRACTION-3 trial were analyzed. The primary endpoint was overall survival (OS). Secondary endpoints included duration of response (DOR), objective response rate (ORR), disease control rate (DCR), and safety. Exploratory subgroup analyses evaluated the association between OS and stratification factors/baseline variables.

**Results:**

Overall, 274 (nivolumab, 136; chemotherapy, 138) of the 419 patients in ATTRACTION-3 were enrolled from Japan: response-evaluable population (107; 108) and safety population (135; 138). OS tended to be longer in the nivolumab group versus the chemotherapy group (median: 13.4 months vs. 9.4 months; HR, 0.77; 95% CI 0.59–1.01). Median DOR was longer in the nivolumab group (7.6 months) versus the chemotherapy group (3.6 months). ORRs were similar between the nivolumab [22.4% of patients (24/107)] and chemotherapy groups [22.2% (24/108); odds ratio, 0.98; 95% CI 0.52–1.87]. DCR was lower in the nivolumab group [41.1% (44/107)] versus the chemotherapy group [66.7% (72/108)]. OS in the exploratory analysis consistently favored the nivolumab group versus the chemotherapy group. Overall, nivolumab demonstrated favorable efficacy and safety versus chemotherapy in the Japanese subpopulation, and the trend was similar to that observed in the overall ATTRACTION-3 ITT population.

**Conclusion:**

Nivolumab represents a new standard second-line treatment option for Japanese patients with advanced ESCC.

**Electronic supplementary material:**

The online version of this article (10.1007/s10388-020-00794-x) contains supplementary material, which is available to authorized users.

## Introduction

Metastatic esophageal cancer has a poor prognosis, with a 5-year relative survival rate of < 8% globally, including in Japan [1, 2]. Esophageal squamous cell carcinoma (ESCC) is the dominant histological subtype worldwide (~ 90%) [3, 4]. Furthermore, differences in baseline characteristics are observed between Japanese and Western patients with esophageal cancer; the incidence of squamous cell carcinoma is higher in Japanese patients than in Western patients [5]. In Japan, fluoropyrimidine plus platinum compounds are used as first-line therapy and taxanes as second-line therapy in patients with unresectable esophageal cancer [6, 7].

Globally, current second-line chemotherapy options for ESCC offer poor long-term survival [7–12]. Therefore, new therapeutic approaches are warranted for patients with advanced ESCC. Immune checkpoint inhibition is one such strategy successfully evaluated in many cancers. The efficacy and safety of nivolumab (an immune checkpoint inhibitor [ICI]) has been demonstrated in esophageal cancer, with a favorable 2-year overall survival (OS) in Japanese patients (17.2%) in the phase 2, single-arm ATTRACTION-1 trial [12, 13] and with significant prolongation of OS [median OS, 10.9 months vs. 8.4 months; hazard ratio (HR) for death, 0.77; *p* = 0.019] in the global, phase 3, randomized, ATTRACTION-3 trial compared with chemotherapy [14]. A smaller proportion of patients in the nivolumab group [38/209 (18%)] experienced grade 3/4 treatment-related adverse events (TRAEs) versus those in the chemotherapy group [131/208 (63%)] in ATTRACTION-3 [14].

Based on ATTRACTION-1 and ATTRACTION-3 results, nivolumab was approved in Japan for the treatment of patients with unresectable advanced or recurrent esophageal cancer on February 21, 2020 [15]. The Japanese esophageal cancer practice guidelines state that second-line chemotherapy for Japanese patients with esophageal cancer has been evaluated in a small sample size in phase 2 studies only, with no evidence of clear efficacy from any reports [6, 7]. Therefore, it is meaningful to evaluate the efficacy and safety of nivolumab in the Japanese population. Hence, this subgroup analysis was performed to evaluate the efficacy and safety of nivolumab versus chemotherapy in the Japanese subpopulation, and to assess whether the results in the Japanese population are similar to those in the overall intent-to-treat (ITT) population of ATTRACTION-3, which was conducted in patients with advanced ESCC [14].

## Methods

### Study design and patients

Data from Japanese patients enrolled in the multicenter, randomized, open-label, phase 3 ATTRACTION-3 trial were analyzed. The results were compared and discussed with the outcomes from the overall study population (enrolled at 90 hospitals across Denmark, Germany, Italy, Japan, South Korea, Taiwan, the UK, and the USA), with November 12, 2018, as the data cutoff date for comparison. The study design and overall results have been previously reported [14]. Briefly, patients aged ≥ 20 years with unresectable esophageal cancer whose major current or previously resected lesion was in the cervical or thoracic esophagus (including the esophagogastric junction) and was pathologically confirmed as squamous or adenosquamous cell carcinoma were enrolled. Eligible patients were refractory or intolerant to fluoropyrimidine- and platinum-based chemotherapy, had previously received one treatment regimen, were not indicated for a radical resection, and had a life expectancy of ≥ 3 months.

Totally, 419 patients were enrolled in the overall study population and randomly assigned (1:1) to receive nivolumab (*n* = 210) or the investigator’s choice of chemotherapy [paclitaxel (PTX) or docetaxel (DTX), *n* = 209]. Randomization was performed using an interactive web response system (block size = 4), and patients were stratified according to their geographical location (Japan vs the rest of the world), number of organs with metastases (≤ 1 vs. ≥ 2), and expression of programmed death-ligand 1 (PD-L1: < 1% vs. ≥ 1%).

The study was performed in accordance with the Good Clinical Practice guidelines developed by the International Council for Harmonisation and approved by the institutional review board or independent ethics committee at each study site. All patients provided written informed consent before enrollment.

### Treatment

Nivolumab was administered at 240 mg intravenously (IV) over 30 min every. 2 weeks (each cycle was 6 weeks long). PTX was administered at 100 mg/m^2^ IV for  ≥ 60 min once per week for 6 weeks followed by 1 treatment-free week (each cycle was 7 weeks long), and DTX was administered at 75 mg/m^2^ IV for ≥ 60 min every 3 weeks (each cycle was 3 weeks long), until disease progression or unacceptable toxicity.

Treatment that was interrupted or delayed due to adverse events (AEs) was resumed when patients met the protocol-defined criteria for treatment resumption. Per protocol, pre-specified dose reductions were permitted for toxicities related to PTX and DTX. Dose reductions were prohibited in the nivolumab group. Additional details pertaining to study procedure have been reported previously [14].

### Assessments

Tumor assessments were performed using computed tomography or magnetic resonance imaging per Response evaluation criteria in solid tumors (RECIST) version 1.1 at baseline, after each 6-week cycle for 1 year, and every 12 weeks thereafter until initiation of post-study treatment, disease progression, or recurrence. Complete response (CR) and partial response (PR) were confirmed by ≥ 2 successive scans within a minimum of 4 weeks. Tumor cell PD-L1 expression was assessed by a central laboratory using immunohistochemistry (PD-L1 IHC 28–8 pharmDx assay; Dako, an Agilent Technologies company, Santa Clara, CA, USA).

Exploratory subgroup analyses evaluated the association between OS and stratification factors or baseline variables. The pre-specified exploratory endpoint assessed the health-related quality of life (HRQoL) based on the three-level version of the EuroQol 5D questionnaire (EQ-5D-3L).

AEs were assessed according to the National Cancer Institute Common Terminology Criteria for Adverse Events (CTCAE) version 4.0 throughout the treatment period and for 28 days after the end of treatment. Serious AEs were assessed throughout the study period and for 100 days after treatment discontinuation. Other AEs data were collected for 28 days after treatment discontinuation.

### Outcomes

The primary endpoint was OS. Secondary endpoints included the proportion of patients with an investigator-assessed objective response rate [ORR; the percentage of patients whose best overall response (BOR) was either a CR or a PR], BOR, progression-free survival (PFS), proportion of patients with disease control [CR + PR + stable disease (SD)], maximum percentage change from baseline in the sum of target lesion diameters, time to response (time from randomization to the first confirmed CR/PR), duration of response (DOR; time from the first response to the first documented tumor progression or death), and safety. Pre-specified exploratory subgroup analyses assessed the association between OS and stratification factors or baseline variables, including PD-L1 expression (< 1%, ≥ 1%, < 5%, ≥ 5%, < 10%, and ≥ 10%), age (< 65 years vs. ≥ 65 years), sex, race (Asian vs white), Eastern Cooperative Oncology Group (ECOG) performance status (PS; 0 vs. 1), prior surgery, prior radiotherapy, and history of smoking. As a pre-specified exploratory endpoint, HRQoL was assessed based on EQ-5D-3L, comprising the visual analog scale (VAS) and descriptive system, to generate the utility index. Assessments were performed every 6 weeks from the start of cycle 1 until the end of the treatment phase and every 12 weeks thereafter. Further details have been reported previously [14].

### Statistical analysis

OS and PFS analyses were performed in the Japanese subpopulation, defined as all patients who were randomly assigned to the study treatment. ORR, BOR, disease control rate (DCR), time to response, and DOR were assessed in all randomized patients in the Japanese subpopulation who had target lesion measurements at baseline (i.e., the response-evaluable population). Safety was assessed in all patients in the Japanese subpopulation who received ≥ 1 dose of the assigned treatment (safety population). Both descriptive and mixed-effect model for repeated measure (MMRM) analyses of patient-reported outcomes were performed for all randomized patients in the Japanese subpopulation who had an EQ-5D-3L and utility index assessment at baseline and ≥ 1 post-baseline assessment including unscheduled or follow-up visits (i.e., patient-reported outcomes population). Estimates of median OS, PFS, and DOR were derived from the Kaplan–Meier (KM) estimates, and the corresponding two-sided 95% confidence intervals (CIs) were calculated using the Brookmeyer and Crowley method based on a log–log transformation. The stratified Cox proportional hazards regression model, with randomization factors as stratification factors and treatment group as a single covariate, was used to assess differences between the treatment groups for OS and PFS; a two-sided stratified log-rank test using randomization stratification factors with a 5% significance level was used. Further details are presented as footnotes or have been published previously [14]. Statistical analyses were performed using the SAS software (version 9.4; SAS Institute, Cary, NC, USA).

## Results

Overall, 274 (nivolumab, 136; chemotherapy, 138) of the 419 patients in ATTRACTION-3 were enrolled from 45 study sites in Japan. The response-evaluable population comprised 215 patients (nivolumab, 107; chemotherapy, 108); the safety population comprised 273 patients (nivolumab, 135; chemotherapy, 138). At database lock (November 12, 2018), the minimum follow-up period was 17.6 months. Baseline characteristics of the patients were well balanced between the treatment groups (Table 1). Overall, 83.0% and 27.5% of patients received 90% to < 110% of nivolumab and chemotherapy planned relative dose intensity, respectively, in the Japanese subpopulation (Supplementary Table 1).

**Table 1:** Patient demographics and baseline characteristics

Trial	Japanese subpopulation	
Characteristic, *n* (%)	Nivolumab *n* = 136	Chemotherapy *n* = 138
Age, median (range), years	65.0 (41–82)	68.0 (33–80)
Age ≥ 65 years	76 (55.9)	98 (71.0)
Sex, male	113 (83.1)	117 (84.8)
ECOG PS 0	83 (61.0)	88 (63.8)
ECOG PS 1	53 (39.0)	50 (36.2)
Recurrent EC	65 (47.8)	70 (50.7)
Number of organs with metastases (IWRS source)		
≤ 1	65 (47.8)	66 (47.8)
≥ 2	71 (52.2)	72 (52.2)
Prior surgery	68 (50.0)	69 (50.0)
Prior radiotherapy	99 (72.8)	89 (64.5)
PD-L1 expression		
≥ 10%	41 (30.1)	35 (25.4)
≥ 5%	46 (33.8)	46 (33.3)
≥ 1%	66 (48.5)	68 (49.3)

*EC* esophageal cancer; *ECOG PS* Eastern Cooperative Oncology Group performance status; *IWRS* interactive web response system; *PD-L1* programmed death-ligand 1

At database lock, study treatment was permanently discontinued in 124/135 (91.9%) patients in the nivolumab group and 135/138 (97.8%) patients in the chemotherapy group in the Japanese subpopulation; reasons for treatment discontinuation (nivolumab vs chemotherapy) were disease progression [86 (63.7%) vs. 98 (71.0%)], worsening of clinical symptoms judged as progressive disease [PD; 15 (11.1%) vs. 8 (5.8%)], onset of grade ≥ 2 interstitial lung disease [9 (6.7%) vs. 4 (2.9%)], treatment withheld for > 6 weeks due to AEs [3 (2.2%) vs. 2 (1.4%)], onset of grade ≥ 3 peripheral neuropathy [0 (0%) vs. 2 (1.4%)], drug-related liver function test abnormality [1 (0.7%) vs 0 (0%)], onset of grade ≥ 3 hypersensitivity (eg, diarrhea, colitis, neurologic toxicity, hypersensitivity reaction, infusion reaction) [1 (0.7%) vs. 0 (0%)], three rounds of dose reductions [0 (0%) vs. 4 (2.9%)], physician’s discretion [9 (6.7%) vs. 13 (9.4%)], and other reasons [5 (3.7%) vs. 8 (5.8%)]. After study treatment discontinuation, the proportion of patients in the Japanese subpopulation who received subsequent anticancer treatment was higher in the nivolumab group [58.8% (80/136)] than in the chemotherapy group [47.1% (65/138)]. The proportion of patients receiving taxanes as subsequent anticancer therapy in the nivolumab group was high in the Japanese subpopulation [56.6% (77/136); Supplementary Table 2]. Further details of subsequent anticancer treatment in the Japanese subpopulation are presented in Supplementary Table 2.

### Efficacy in the Japanese subpopulation

#### Overall survival

**Fig. 1:**
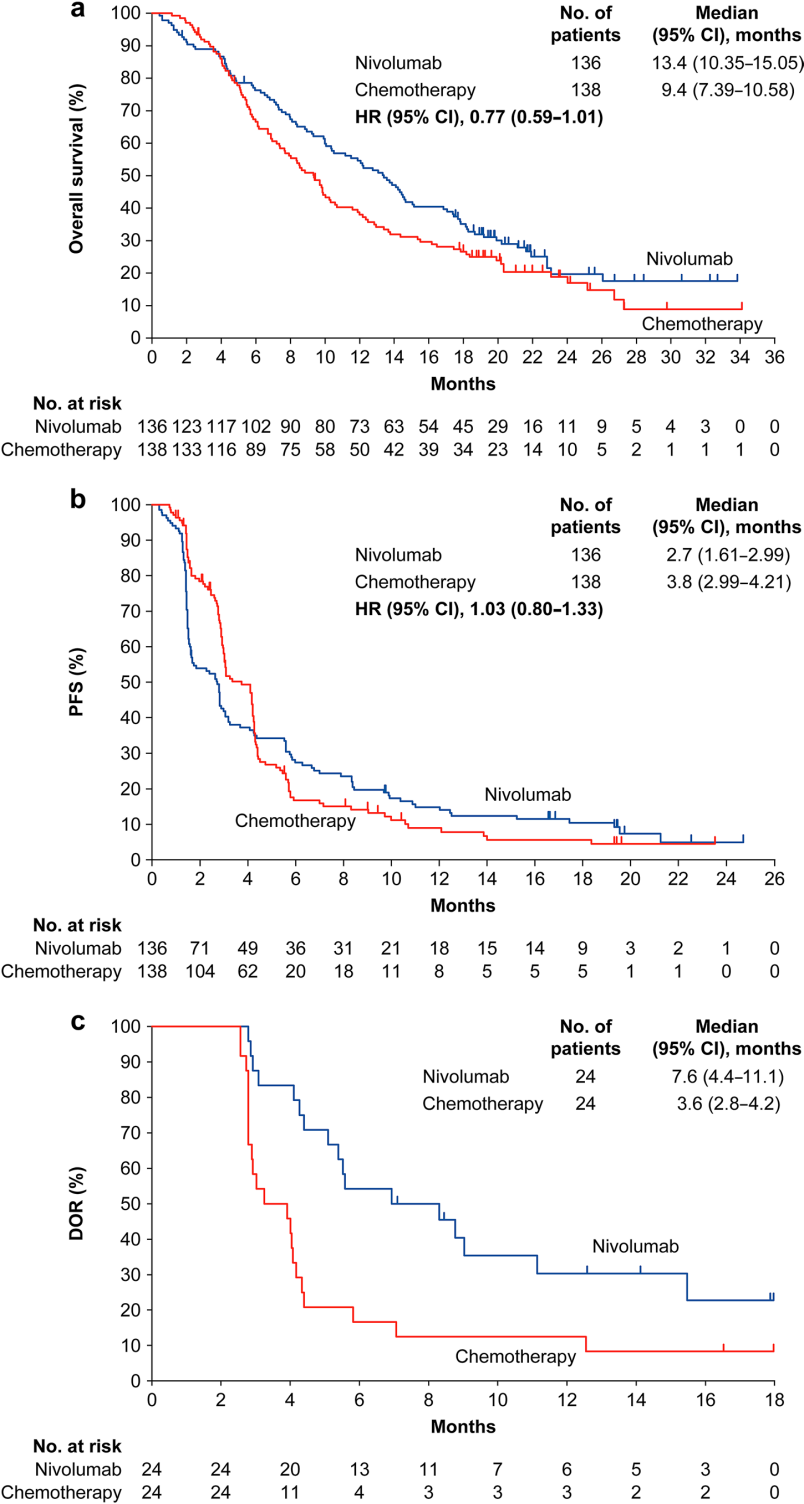
Kaplan–Meier plots of **a** overall survival, **b** PFS, and **c** DOR in the Japanese subpopulation**.** *CI* confidence interval, *DOR* duration of response, *HR* hazard ratio, *PFS* progression-free survival

**Fig. 2:**
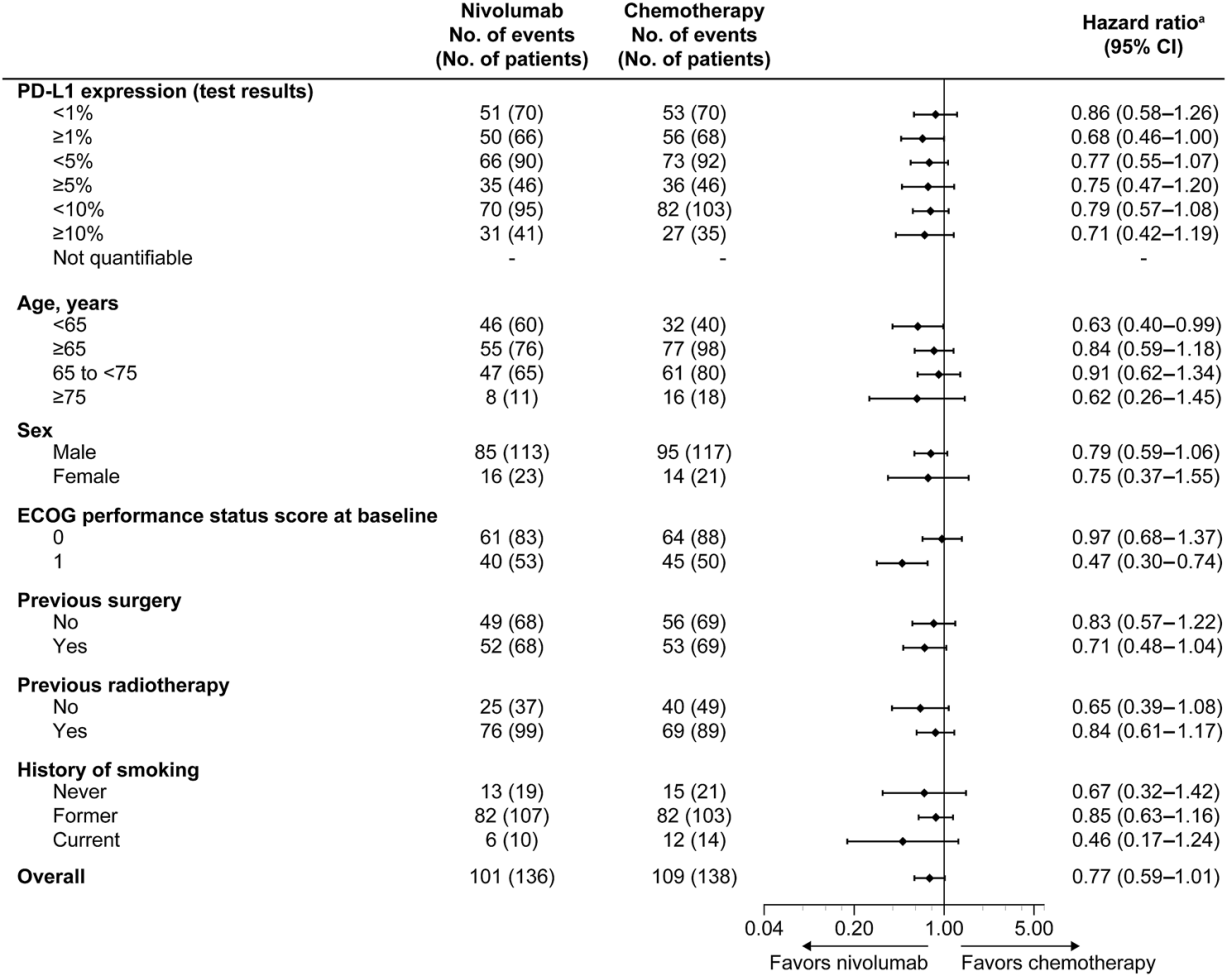
Forest plot of overall survival according to patient subgroups (Japanese subpopulation). ^a^Hazard ratios and their corresponding 95% CIs for nivolumab relative to chemotherapy were calculated using the unstratified Cox proportional hazards model. *CI* confidence interval, *ECOG* Eastern Cooperative Oncology Group, *PD-L1* programmed death-ligand 1

Median follow-up was 13.21 months [interquartile range (IQR), 6.11–19.48; *n* = 136] in the nivolumab group and 8.74 months (IQR, 5.06–17.84; *n* = 138) in the chemotherapy group. By KM analysis, OS was numerically longer in the nivolumab group versus the chemotherapy group [median: 13.4 months vs. 9.4 months; HR, 0.77 (95% CI 0.59–1.01); Fig. 1a]. In the subgroup analysis, OS was numerically longer in the nivolumab group consistently versus the chemotherapy group in the Japanese subpopulation (Fig. 2 and Supplementary Fig. 1). In the Japanese ITT subpopulation, as of November 2018, five deaths had occurred due to TRAEs (nivolumab, 2/135; chemotherapy, 3/138).

#### Progression-free survival

By KM analysis, the median PFS was 2.7 months (95% CI 1.61–2.99) in the nivolumab group versus 3.8 months (95% CI 2.99–4.21) in the chemotherapy group (HR, 1.03; 95% CI 0.80–1.33; Fig. 1b).

#### Response

Patients with confirmed CR or PR to nivolumab showed a median time to response of 2.74 months (minimum–maximum, 1.3–6.5). The median DOR was numerically longer in the nivolumab group (7.6 months; 95% CI 4.4–11.1) versus the chemotherapy group (3.6 months; 95% CI 2.8–4.2; Fig. 1c). ORRs were similar between the nivolumab [22.4% (24/107)] and chemotherapy groups [22.2% (24/108); odds ratio, 0.98; 95% CI 0.52–1.87]. DCR was lower in the nivolumab group [41.1% (44/107)] versus the chemotherapy group [66.7% (72/108); odds ratio, 0.30; 95% CI 0.17–0.55; Table 2 a].

**Table 2:** Response and disease control (**a**) and summary of treatment-related adverse events (**b**) in the Japanese subpopulation

*n* (%)	Japanese subpopulation (RES)		
	Nivolumab (*n* = 107)	Chemotherapy (*n* = 108)	OR (95% CI)
**(a) Response and disease control**			
ORR	24 (22.4)	24 (22.2)	0.98 (0.52–1.87)
CR	0	2 (1.9)	
PR	24 (22.4)	22 (20.4)	
SD	20 (18.7)	48 (44.4)	
PD	58 (54.2)	32 (29.6)	
NE	5 (4.7)	4 (3.7)	
DCR (CR + PR + SD)	44 (41.1)	72 (66.7)	0.30 (0.17–0.55)

*n* (%)	Nivolumab (*n* = 135)				Chemotherapy (*n* = 138)			
	Grade 1/2	Grade 3	Grade 4	Grade 5	Grade 1/2	Grade 3	Grade 4	Grade 5
**(b) Summary of treatment-related adverse events**								
All events	69 (51)	20 (15)	3 (2)	0	33 (24)	66 (48)	34 (25)	2 (1)
Serious events	9 (7)	12 (9)	2 (1)	0	5 (4)	21 (15)	5 (4)	2 (1)
Events leading to discontinuation	9 (7)	7 (5)	0	0	5 (4)	7 (5)	2 (1)	1 (< 1)
Events leading to death	0	2 (1)	0	0	0	1 (< 1)	0	2 (1)
Events in ≥ 10% of treated patients in either group								
Rash	15 (11)	0	0	0	18 (13)	0	0	0
Hypothyroidism	14 (10)	0	0	0	0	0	0	0
Pyrexia	10 (7)	1 (< 1)	0	0	15 (11)	0	0	0
Diarrhoea	8 (6)	1 (< 1)	0	0	12 (9)	2 (1)	0	0
Fatigue	8 (6)	0	0	0	21 (15)	4 (3)	0	0
Malaise	8 (6)	0	0	0	42 (30)	0	0	0
Decreased appetite	7 (5)	0	0	0	32 (23)	7 (5)	0	0
Stomatitis	4 (3)	0	0	0	20 (14)	1 (< 1)	0	0
Dysgeusia	3 (2)	0	0	0	14 (10)	0	0	0
Lymphocyte count decreased	2 (1)	2 (1)	0	0	6 (4)	9 (7)	3 (2)	0
Alopecia	2 (1)	0	0	0	82 (59)	0	0	0
Arthralgia	2 (1)	0	0	0	16 (12)	0	0	0
Constipation	2 (1)	0	0	0	14 (10)	0	0	0
Neutrophil count decreased	2 (1)	0	0	0	13 (9)	27 (20)	26 (19)	0
White blood cell count decreased	1 (< 1)	1 (< 1)	0	0	18 (13)	29 (21)	13 (9)	0
Nausea	1 (< 1)	0	0	0	22 (16)	0	0	0
Neutropenia	1 (< 1)	0	0	0	6 (4)	16 (12)	6 (4)	0
Peripheral sensory neuropathy	1 (< 1)	0	0	0	42 (30)	1 (< 1)	0	0
Anaemia	0	3 (2)	0	0	20 (14)	16 (12)	0	0
Febrile neutropenia	0	0	0	0	0	14 (10)	1 (< 1)	0
Neuropathy peripheral	0	0	0	0	15 (11)	0	0	0

The deaths were caused by interstitial lung disease and pneumonitis in the nivolumab group and by pneumonia, spinal cord abscess, and interstitial lung disease in the chemotherapy group. Some patients had adverse events lower than grade 5 that subsequently led to death

*CI* confidence interval, *CR* complete response, *DCR* disease control rate, *NE* not evaluable, *OR* odds ratio, *ORR* objective response rate, *PD* progressive disease, *PR* partial response, *RES* response-evaluable set, *SD* stable disease

#### Tumor burden

The best change from baseline in target lesion size in the nivolumab group versus the chemotherapy group is presented in Supplementary Fig. 2.

#### PD-L1 expression status

Baseline tumor samples for determination of PD-L1 were available for all patients in the Japanese subpopulation. The median OS in patients with < 1% versus ≥ 1% tumor PD-L1 expression was 13.4 months (95% CI 8.84–17.05) versus 12.75 months (9.92–17.84), respectively, with nivolumab and 10.32 months (7.39–13.67) versus 8.38 months (5.98–9.89), respectively, with chemotherapy. The pre-specified interaction analysis indicated no significant interaction of treatment effect by PD-L1 status in the Japanese subpopulation (Supplementary Fig. 3). The median OS in the Japanese subpopulation based on tumor PD-L1 expression (< 1% vs. ≥ 1%) was similar to that in the overall ITT population.

#### Quality of life

The proportion of patients completing the EQ-5D-3L questionnaires exceeded 87% in both groups through week 42. In the Japanese subpopulation, the on-treatment improvement in quality of life (QoL) of patients was more favorable in the nivolumab group than in the chemotherapy group (Supplementary Fig. 4). This improvement in QoL was similar to that observed in the overall ITT population [14].

### Safety

TRAEs of any grade and grade 3–5 were observed in a lower proportion of patients in the nivolumab group (68.1%, 17.0%) versus the chemotherapy group (97.8%, 73.9%). Events in ≥ 10% of the treated patients in either group are listed in Table 2 b.

## Discussion

To date, ICIs have been evaluated in patients with advanced esophageal cancer in two global studies (ATTRACTION-3 [14] and KEYNOTE-181 [16]). Of these studies, the proportion of Japanese patients was higher in ATTRACTION-3 [65.4% (274/419)] than in KEYNOTE-181 [24.2% (152/628)]. This sub-analysis of ATTRACTION-3 [14] is the first report demonstrating the efficacy and safety of nivolumab in a phase 3 trial in patients with esophageal cancer in Japan.

The baseline patient characteristics were mostly similar between the Japanese subpopulation and the overall ITT population [14]. However, more patients in the Japanese subpopulation had an ECOG PS of 0 in both groups versus the overall ITT population [61.0% (83/136) vs. 48.1% (101/210)]. Median OS for nivolumab was numerically longer in the Japanese subpopulation (13.4 months) versus the overall ITT population (10.9 months); a similar trend in OS was also observed in the chemotherapy group (9.4 months vs. 8.4 months). In the subgroup analysis, OS was consistently numerically longer in the nivolumab group versus the chemotherapy group in the Japanese subpopulation, which was similar to the trend observed in the overall ITT population [14]. Further, the median PFS, ORR, and DCR in the Japanese subpopulation were numerically similar to those in the overall ITT population [14].

This trend in the improvement in OS may be attributed to a higher proportion of patients reporting activities of daily living and physical ability (ECOG PS, 0) in the Japanese subpopulation versus the overall ITT population. This trend in OS was also observed in nivolumab trials conducted in advanced melanoma and non-small cell lung cancer [17, 18]. Patients with a good PS/clinical condition are also likely to receive subsequent treatment.

To this end, we observed differences in subsequent anticancer therapy that may have affected the results. After study treatment discontinuation, the proportion of patients who received subsequent anticancer treatment in the Japanese subpopulation and the overall ITT population was numerically higher in the nivolumab group [58.8% (80/136) and 53% (112/210)] than in the chemotherapy group [47.1% (65/138) and 47% (99/209)]. Furthermore, the proportion of patients receiving taxanes as subsequent anticancer therapy in the nivolumab group was higher in the Japanese subpopulation versus the overall ITT population [56.6% (77/136) vs. 48% (100/210)]; 66/75 patients who received PTX as subsequent anticancer therapy were Japanese. The reason for high PTX use in the Japanese population can be explained by the approval status and guideline description of PTX in Japan. PTX has been approved for the treatment of esophageal cancer and described in the guidelines [7] based on the results of a phase 2 study conducted in Japan [8]. Regarding the patients enrolled from other countries in ATTRACTION-3 [14], PTX is not approved in Korea or Taiwan [19], and the proportion of patients from the remaining countries was limited. Therefore, PTX is considered to be more frequently used in the Japanese population than in the ITT population in ATTRACTION-3 and might have influenced OS. ICI treatment followed by chemotherapy reportedly has a favorable outcome [20, 21], suggesting that the improved OS outcomes for nivolumab in the Japanese subpopulation could be attributed to the differences in subsequent anticancer therapies used as third-line treatment. Considering that OS may be indicative of the efficacy of both nivolumab and subsequent therapy, it is hypothesized that OS, and not PFS, is likely to be different between the Japanese population and overall ITT population based on the higher proportion of patients who received taxanes as subsequent therapy in the Japanese subpopulation than in the overall ITT population. Our data, however, showed that the HR for OS was the same in the Japanese subpopulation (0.77) and the overall ITT population (0.77), while the HR for PFS was similar in the Japanese subpopulation (1.03) and the overall ITT population (1.08). Based on these results, the difference in subsequent treatment regimens seems to have little impact on OS in the Japanese population and the overall ITT population. Alternatively, the difference in the proportion between the Japanese population and the overall ITT population is not enough to detect the differential effects of subsequent regimens. Therefore, it may be desirable to evaluate the effect of ICIs on subsequent chemotherapy regimens in the near future.

No notable difference in the efficacy and safety of nivolumab was observed between the Japanese subpopulation and the overall ITT population enrolled in ATTRACTION-3 [14]. Similar subgroup analyses in Japanese or Asian subpopulations who received nivolumab for other cancers were consistent with those reported for the overall ITT population [22–24].

## Conclusion

In the Japanese subpopulation, the OS was numerically longer for nivolumab versus chemotherapy, which was similar to the trend observed in the overall ITT population. The frequency of PTX use in the post-study treatment was different between the Japanese subpopulation and the overall ITT population, but this difference in the treatment environment was not clearly reflected in the study results. Additionally, no notable difference was observed between the safety profiles of the Japanese subpopulation and those of the overall ITT population. Nivolumab represents a new standard second-line treatment option for Japanese patients with advanced ESCC refractory to prior fluoropyrimidine-based chemotherapy.

## Electronic supplementary material

Below is the link to the electronic supplementary material.

Supplementary file1 (PDF 964 KB)
