# Supplementary material for: Nivolumab versus chemotherapy in Japanese patients with advanced esophageal squamous cell carcinoma: a subgroup analysis of a multicenter, randomized, open-label, phase 3 trial (ATTRACTION-3)
Source: Esophagus. 2020 Nov 10;18(1):90–9. doi: 10.1007/s10388-020-00794-x (PMC7794205; doi:10.1007/s10388-020-00794-x)
Supplement: Supplementary file 1 — Supplementary file1 (PDF 964 KB) [file 10388_2020_794_MOESM1_ESM.pdf]

SUPPLEMENTARY DATA

Target journal: *Esophagus*

Title: Nivolumab versus chemotherapy in Japanese patients with advanced esophageal squamous cell carcinoma: a subgroup analysis of a multicenter, randomized, open-label, phase 3 trial (ATTRACTION-3)

Authors: Masanobu Takahashi[1], Ken Kato[2], Morihito Okada[3], Keisho Chin[4], Shigenori Kadowaki[5], Yasuo Hamamoto[6], Yuichiro Doki[7], Yutaro Kubota[8], Hisato Kawakami[9], Takashi Ogata[10], Hiroki Hara[11], Manabu Muto[12], Yuichiro Nakashima[13], Ryu Ishihara[14], Masahiro Tsuda[15], Satoru Motoyama[16], Mamoru Kodani[17], Yuko Kitagawa[18]

Affiliations:

[1]Department of Medical Oncology, Tohoku University Hospital, Miyagi, Japan

[2]Department of Gastrointestinal Medical Oncology, National Cancer Center Hospital, Tokyo, Japan

[3]Department of Surgical Oncology, Research Institute for Radiation Biology and Medicine, Hiroshima University, Hiroshima, Japan

[4]Department of Gastroenterological Chemotherapy, The Cancer Institute Hospital of Japanese Foundation for Cancer Research, Tokyo, Japan

[5]Department of Clinical Oncology, Aichi Cancer Center Hospital, Nagoya, Japan

[6]Keio Cancer Center, Keio University School of Medicine, Tokyo, Japan

[7]Department of Surgery, Osaka University Hospital, Osaka, Japan

[8]Department of Medicine, Division of Medical Oncology, Showa University School of Medicine, Tokyo, Japan

[9]Department of Medical Oncology, Kindai University Faculty of Medicine, Osakasayama, Osaka, Japan

[10]Department of Gastrointestinal Surgery, Kanagawa Cancer Center, Yokohama, Japan

[11]Department of Gastroenterology, Saitama Cancer Center, Saitama, Japan

[12]Department of Clinical Oncology, Kyoto University Hospital, Kyoto, Japan

[13]Department of Surgery and Science, Graduate School of Medical Sciences, Kyushu University, Fukuoka, Japan

[14]Department of Gastrointestinal Oncology, Osaka International Cancer Institute, Osaka, Japan

[15]Department of Gastroenterological Oncology, Hyogo Cancer Center, Akashi, Japan

[16]Department of Thoracic Surgery, Akita University Graduate School of Medicine, Akita, Japan

[17]Department of Oncology, Ono Pharmaceutical Co., Ltd., Osaka, Japan

[18]Department of Surgery, Keio University School of Medicine, Tokyo, Japan

Corresponding author:

Dr. Masanobu Takahashi

Department of Medical Oncology, Tohoku University Hospital

1-1 Seiryo-machi, Aoba-ku, Sendai-shi, Miyagi 980-8574, Japan

Telephone number: 022-717-8543

Fax number: 022-717-8548

Email address: masanobu.takahashi.a7@tohoku.ac.jp

Supplementary Table 1 Study drug exposure and administration status in the Japanese subpopulation

**Analysis item (unit)**	**Nivolumab**<br>**n (%)**<br>**N=135**	**Chemotherapy**<br>**n (%)**<br>**N=138**
Duration of treatment (months)[a]		
>6	37 (27.4)	18 (13.0)
>12	14 (10.4)	6 (4.3)
Mean (SD)	5.1 (5.6)	3.6 (3.7)
Median	2.8	2.6
Min-max	0-23	0-21
Number of cycles[b]		
1	33 (24.4)	30 (21.7)
2-3	49 (36.3)	62 (44.9)
4-6	27 (20.0)	32 (23.2)
≥7	26 (19.3)	14 (10.1)
Mean (SD)	4.2 (4.0)	3.6 (3.4)
Median	3.0	3.0
Min-max	1-17	1-22
Relative dose intensity (%)		
<50	0	5 (3.6)
50 to <70	1 (0.7)	37 (26.8)
70 to <90	21 (15.6)	58 (42.0)
90 to <110	112 (83.0)	38 (27.5)
≥110	1 (0.7)	0
Mean (SD)	95.6 (7.6)	78.6 (16.0)

Analysis item (unit)	Nivolumab<br>n (%)<br>N=135	Chemotherapy<br>n (%)<br>N=138
Median	100.0	78.4
Min-max	67-112	45-108

max maximum; *min* minimum; *SD* standard deviation.

[a]Duration of treatment (months) = ("date of the last dose"-"date of the first dose" + 1)/30.4375

[b]The cycle number was calculated for the cycle proceeding to the next cycle. The discontinued cycle or the cycle receiving no investigational product was also calculated.

Each treatment cycle consists of the following number of weeks:

Nivolumab: 6 weeks

Docetaxel: 3 weeks

Paclitaxel: administered for 6 weeks and 2 weeks rested

For a completed cycle, the defined dose was calculated as 6 x 100 (mg/m^2). For a discontinued cycle, the defined dose was calculated as follows:

"{[date of the last dose - start date of the discontinued cycle + 7] (days) x 100 (mg/m^2)}/7 (days)"

Supplementary Table 2 Summary of posttreatment therapies in Japanese patients

	**Nivolumab**<br>**n (%)**<br>**N=136**	**Chemotherapy**<br>**n (%)**<br>**N=138**
Patients who received any subsequent therapy	85 (62.5)	77 (55.8)
Patients who received subsequent radiotherapy	19 (14.0)	16 (11.6)
Patients who underwent subsequent surgery	5 (3.7)	9 (6.5)
Patients who received subsequent systemic anticancer therapy	80 (58.8)	65 (47.1)
A sclerosing agent	1 (<1)	0
Talc	1 (<1)	0
Taxanes	77 (56.6)	33 (23.9)
Docetaxel	28 (20.6)	9 (6.5)
Paclitaxel	66 (48.5)	25 (18.1)
Fluoropyrimidine-based chemotherapy	18 (13.2)	32 (23.2)
Gimeracil/oteracil potassium/tegafur	13 (9.6)	27 (19.6)
Fluorouracil	7 (5.1)	7 (5.1)
Tegafur	0	1 (<1)
Capecitabine	0	0
Platinum-based chemotherapy	9 (6.6)	10 (7.2)
Cisplatin	5 (3.7)	3 (2.2)
Nedaplatin	4 (2.9)	8 (5.8)
Carboplatin	0	0
Oxaliplatin	0	0
Other systemic cancer therapy	3 (2.2)	10 (7.2)
Buparlisib	0	1 (<1)

	Nivolumab	Chemotherapy
	n (%)<br>N=136	n (%)<br>N=138
Doxorubicin hydrochloride	0	1 (<1)
Other	3 (2.2)	8 (5.8)
Immunotherapy	0	4 (2.9)
Lambrolizumab	0	2 (1.4)
Nivolumab	0	2 (1.4)

Supplementary Fig. 1 Forest plot of additional subgroup analysis of overall survival (Japanese subpopulation)

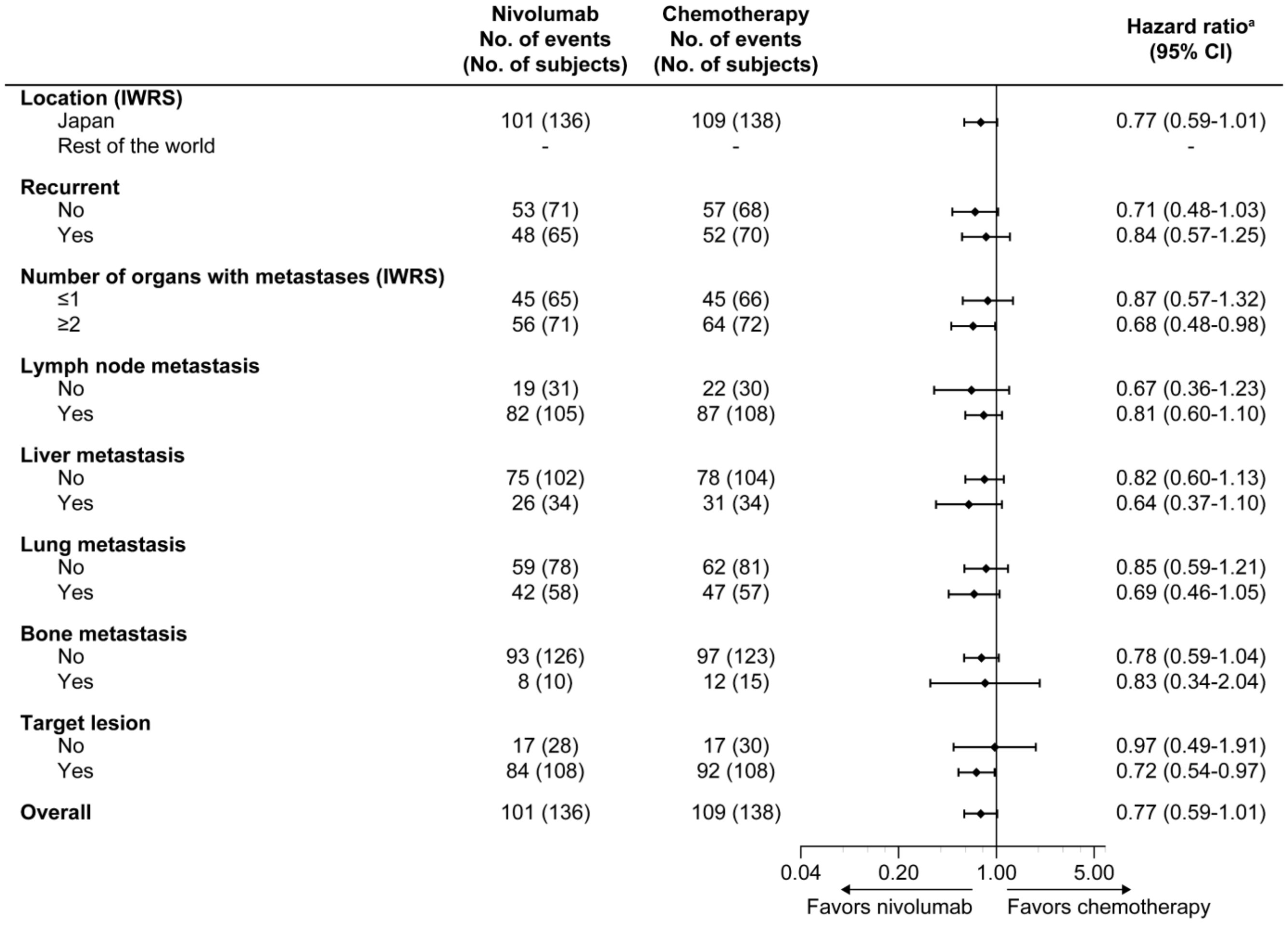

[a]Hazard ratios and their corresponding 95% CIs for nivolumab relative to chemotherapy were calculated using the unstratified Cox proportional hazards model.

CI confidence interval; *IWRS* interactive web response system.

Supplementary Fig. 2 Waterfall plot depicting the best change from baseline in target lesion size per RECIST version 1.1 in patients[a] treated with (a) nivolumab and (b) chemotherapy

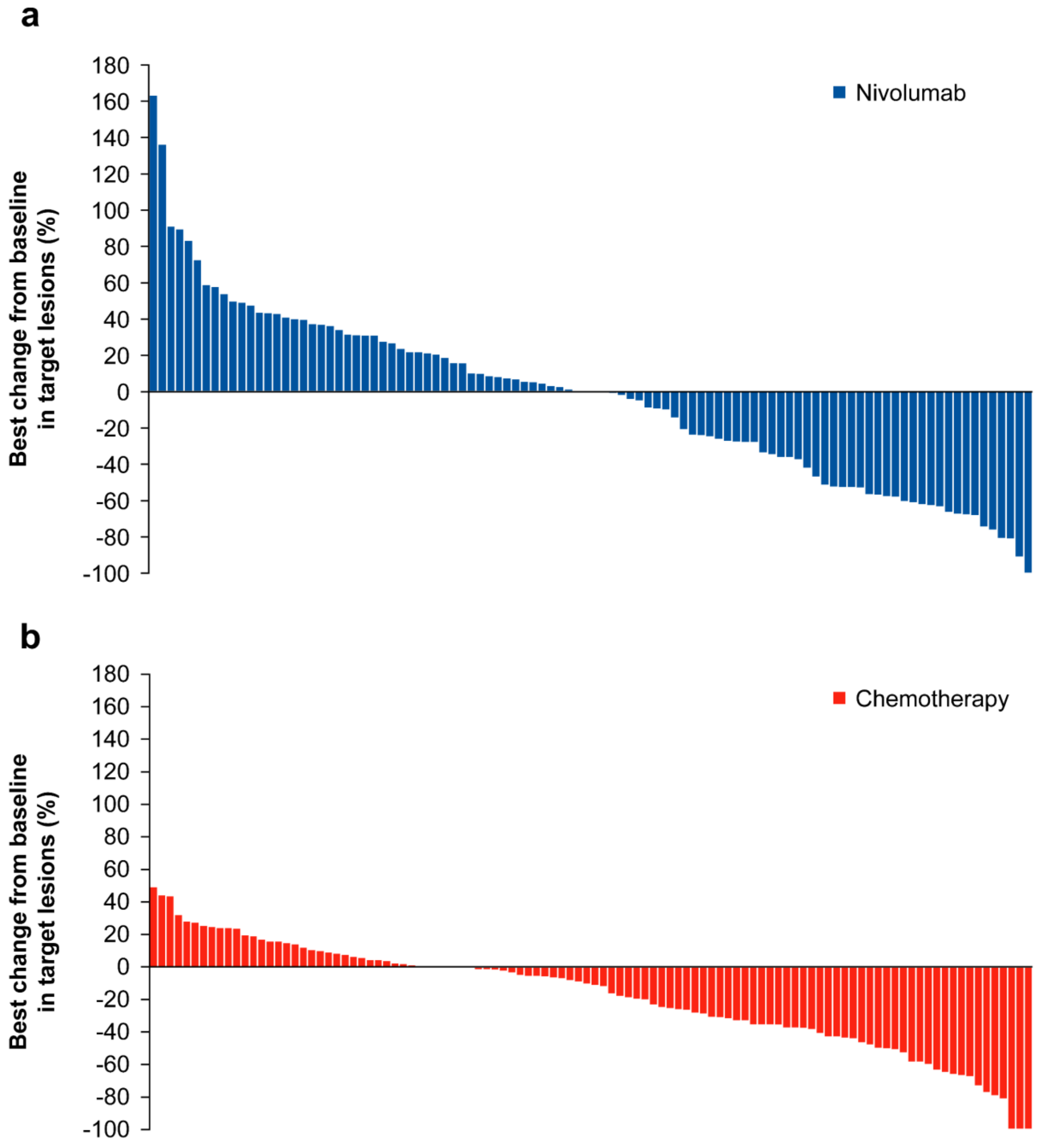

[a]Randomized patients who had target lesion measurements at baseline and were response evaluable.

RECIST Response Evaluation Criteria in Solid Tumors.

Supplementary Fig. 3 Overall survival in patients with tumor PD-L1 expression (a) <1% and (b) ≥1%

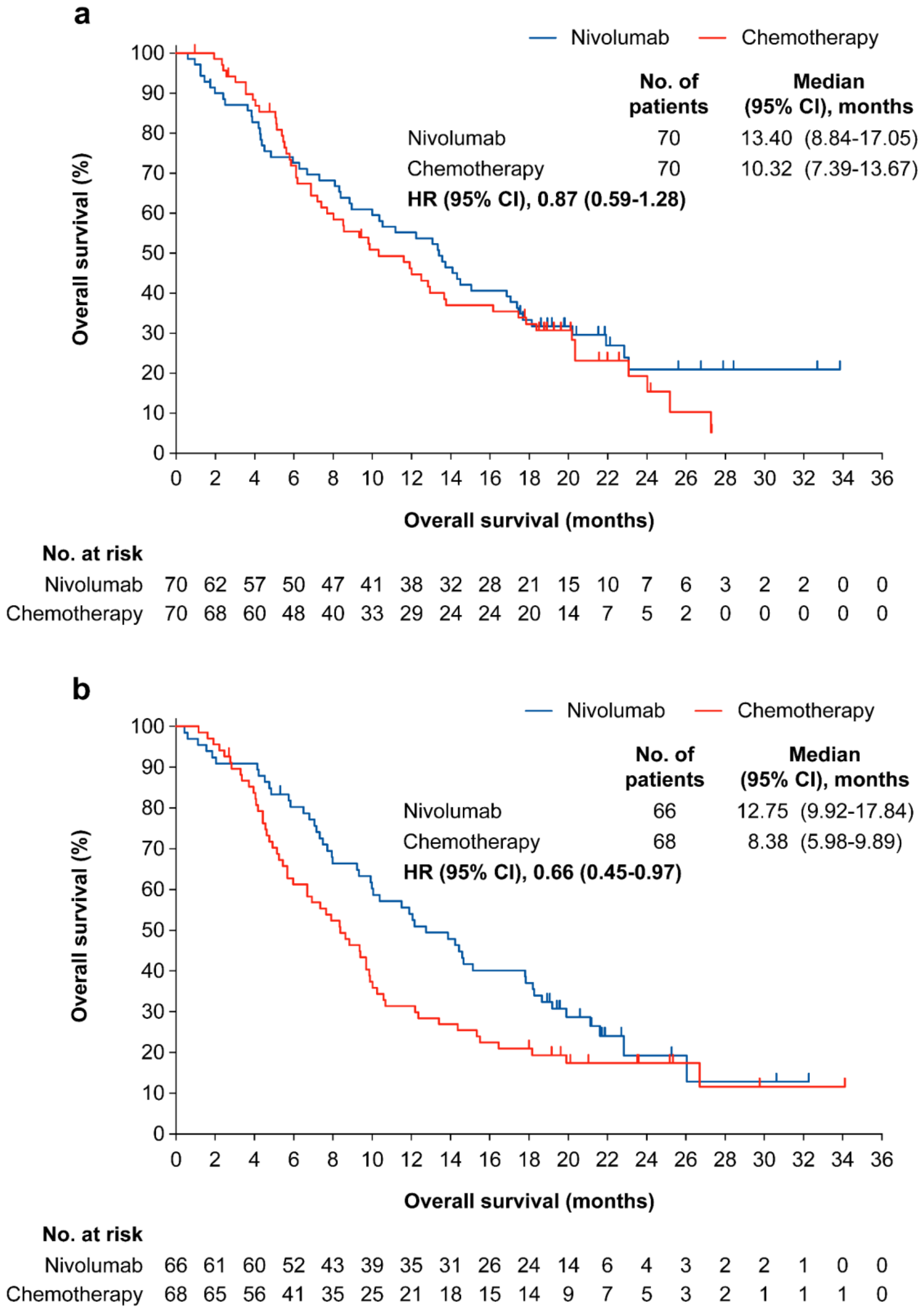

CI confidence interval; *HR* hazard ratio; *PD-L1* programmed death-ligand 1.

Supplementary Fig. 4 EQ-5D-3L VAS change in mean scores (95% CI) across timepoints (a); and LS change means (95% CI) difference between nivolumab and chemotherapy (b); in the patient-reported outcomes population. EQ-5D-3L utility index score change in mean scores (95% CI) across timepoints (c); and LS change means (95% CI) difference between nivolumab and chemotherapy (d); in the patient-reported outcomes population

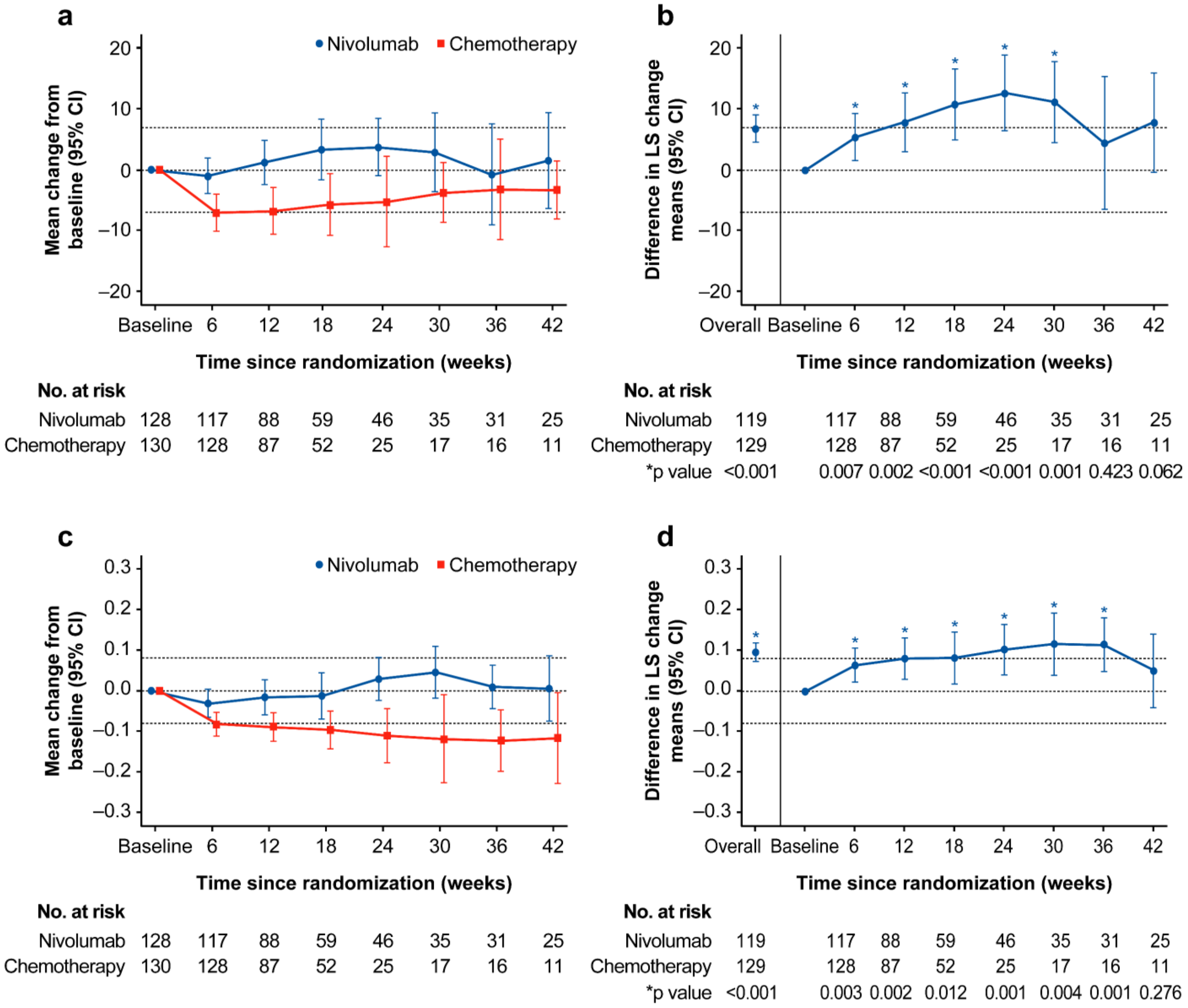

CI confidence interval; *EQ-5D-3L* three-level EuroQol 5D questionnaire; *LS* least squares; *VAS* visual analog scale.

The mean difference between groups favored nivolumab at all timepoints and was clinically meaningful for the VAS from weeks 6 through 30 and for the utility index from weeks 6 through 36.
